# The Transcriptomics of Secondary Growth and Wood Formation in Conifers

**DOI:** 10.1155/2013/974324

**Published:** 2013-10-29

**Authors:** Ana Carvalho, Jorge Paiva, José Louzada, José Lima-Brito

**Affiliations:** ^1^Institute for Biotechnology and Bioengineering, Centre of Genomics and Biotechnology (IBB/CGB), University of Tras-os-Montes and Alto Douro, 5001-801 Vila Real, Portugal; ^2^Instituto de Investigação Científica Tropical (IICT), Centro de Florestas e Produtos Florestais (FLOR), Tapada da Ajuda, 1349-018 Lisboa, Portugal; ^3^Department of Forestry Sciences and Landscape (CIFAP), University of Tras-os-Montes and Alto Douro, 5001-801 Vila Real, Portugal; ^4^Centre for the Research and Technology of Agro-Environmental and Biological Sciences (CITAB), University of Tras-os-Montes and Alto Douro, 5001-801 Vila Real, Portugal

## Abstract

In the last years, forestry scientists have adapted genomics and next-generation sequencing (NGS) technologies to the search for candidate genes related to the transcriptomics of secondary growth and wood formation in several tree species. Gymnosperms, in particular, the conifers, are ecologically and economically important, namely, for the production of wood and other forestry end products. Until very recently, no whole genome sequencing of a conifer genome was available. Due to the gradual improvement of the NGS technologies and inherent bioinformatics tools, two draft assemblies of the whole genomes sequence of *Picea abies* and *Picea glauca* arose in the current year. These draft genome assemblies will bring new insights about the structure, content, and evolution of the conifer genomes. Furthermore, new directions in the forestry, breeding and research of conifers will be discussed in the following. The identification of genes associated with the xylem transcriptome and the knowledge of their regulatory mechanisms will provide less time-consuming breeding cycles and a high accuracy for the selection of traits related to wood production and quality.

## 1. Introduction: The Importance of Xylem Transcriptomics in Gymnosperms

Gymnosperms are seed-bearing plants that include common trees such as pine, spruce, fir, hemlock, and cedar. Among the living gymnosperms, four phyla could be considered: Cycadophyta (cycads), Ginkgophyta (*Ginkgo biloba* L.), Coniferophyta (conifers), and Gnetophyta (gnetophytes) [1]. The conifers are the most numerous gymnosperms, comprising 50 genera and 550 species, and are widely distributed through the northern hemisphere [2]. Pines (family Pinaceae, genus *Pinus*) are among the most economically important conifers, once they constitute the major source of lumber and paper pulp and also have a significant ecological role. Their wide and natural distribution, particularly those from Scots pine (*Pinus sylvestris* L.), reveals high phenotypic plasticity and genetic diversity, providing adaptation to different habitats with variable elevations and climate conditions [3].

The classical goals of breeding programmes in conifers, particularly in pines, are related to the improvement of growth, form, climatic adaptation, disease resistance, variability, and heritability of traits concerned with pulp, paper, and solid-wood end products [2]. The improvement of wood density, stiffness, fibre morphology, and orientation has also been intended in order to achieve high-quality timber-related products. The selection of elite individuals with desirable traits for wood quality in very early stages of tree development constitutes one of the major goals of breeding programmes and forestry industry [4, 5]. Notwithstanding, wood quality is a highly complex trait, and several efforts have been developed in the last years in order to deeply understand the molecular mechanisms underlying it. Technologies such as gene mapping, markers assisted selection (MAS), transcriptome profiling, sequencing, gene silencing, and genetic engineering have been used for the identification and validation of candidate genes related to secondary growth and wood formation in both gymnosperms and angiosperms. Due to the long development cycles of trees until reaching wood production, it was necessary to use nontree model plants for such studies. Given the moderate conservation of the xylem transcriptome among the vascular plants, comparative genomics tools based on known xylem unigenes from *Arabidopsis thaliana* contributed to a deeper understanding of wood formation in trees [6].

Generally, most of the genes differentially expressed in wood-forming tissues are related to the primary and secondary cell wall formation and to the monolignol biosynthesis. Here, we intend to revise some of the last remarkable studies concerning the transcriptomics of secondary growth and wood formation in conifers.

## 2. Wood Formation and Structure

Wood, also known as secondary xylem, derived from the plant secondary growth that occurred at roots and stems due to the activity of highly vacuolated meristematic cells of the vascular and cork cambium (lateral meristems) [1, 7]. Secondary growth involves a sequence of biological events at the cells, including maintenance of division, expansion (elongation and radial enlarging), differentiation, secondary wall thickening (cellulose, hemicellulose, lignin biosynthesis, and deposition), aging, and programmed death [8].

Up to six types of wood can be isolated and studied on a single tree: early wood, late wood, juvenile wood, mature wood, reaction wood, and opposite wood. Each type of wood has different chemical, physical, physiological, mechanical, and anatomical properties [8–10].

The early wood is produced at the beginning of the growing season (spring wood) while late wood is the portion of an annual growth increment produced during the latter part of the growing season (summer wood). Early wood is composed of large diameter cells and has low density, whereas the late wood has smaller diameter cells and high density due to thicker cell walls. A growth ring (ring of wood) easily visible on a cross stem section results from periodic growth. One growth ring formed during a year is called an annual ring.

**Figure 1:**
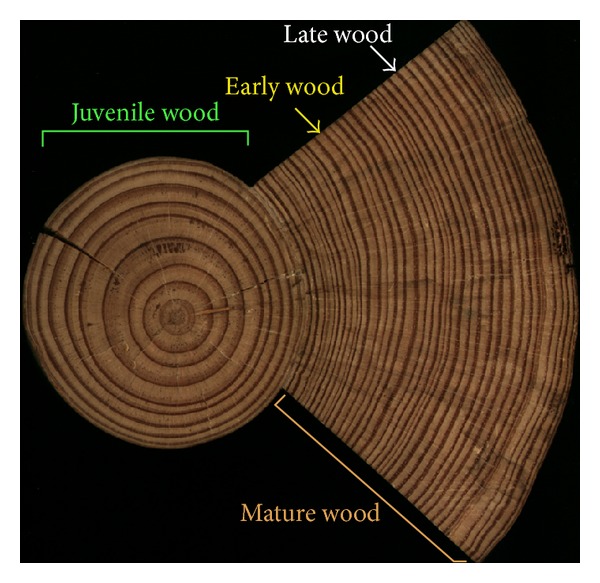
Cross section of a stem from an adult tree of *Pinus pinaster* Ait., showing juvenile and mature wood. The centre of the stem (circular portion at left) has juvenile wood, characterized by growth rings with large width due to a high portion of early wood (lighter rings). Mature wood (external triangular portion at right) presents thin growth rings due to reduced portions of early wood (lighter rings, yellow arrow) and higher percentage of late wood (darker rings, white arrow).

The gradual transition from juvenile to mature wood could not be perceptible in some transverse cuts of hardwood species. However, in softwood species, such as pines, a radial variation could be observed in the wood pattern based on different widths of the growth rings between the juvenile and mature wood [10] (Figure 1).

Usually, juvenile wood of most species has a considerable larger growth ring width than mature wood, and species with fast growth tend to have much higher content of juvenile wood [10]. Juvenile wood is composed of shorter length tracheids (conducting elements) and larger microfibril angles than mature wood [10]. The tracheids of juvenile wood also present smaller radial and tangential diameters and thinner cell walls than mature wood [10], explaining its reduced density compared to mature wood.

**Figure 2:**
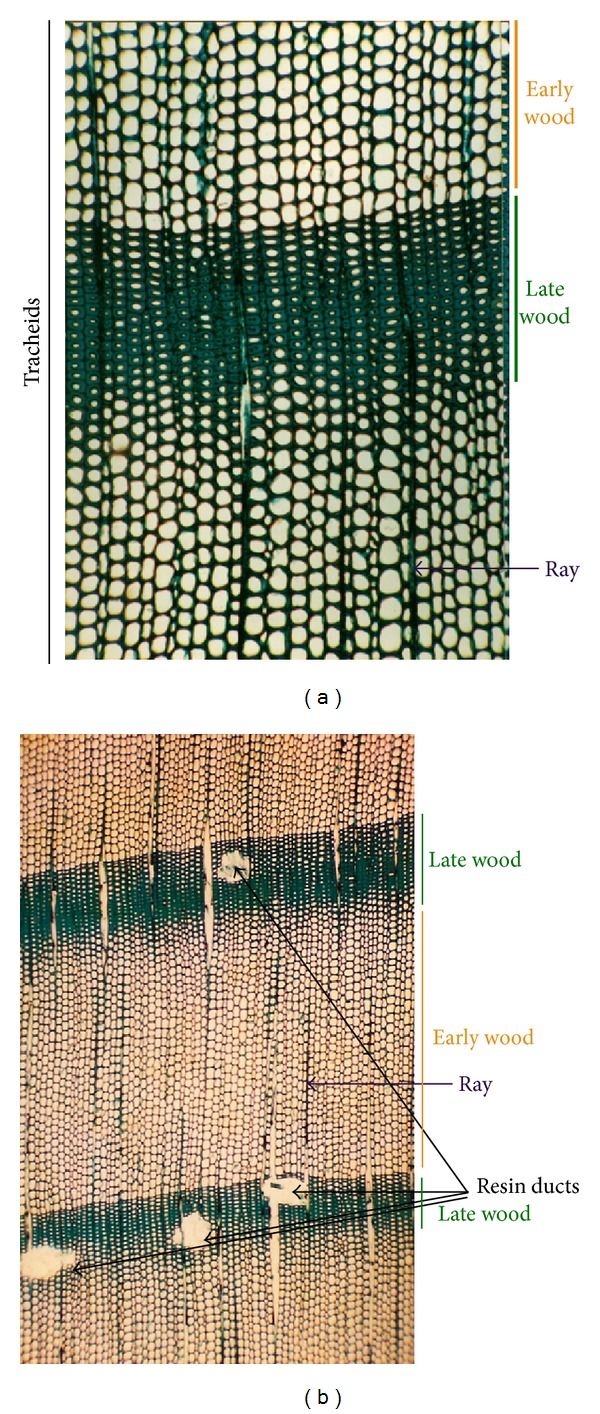
Two histological cuts of mature wood from *P. pinaster* (with different magnifications) showing its simple anatomical structure, mainly composed of longitudinal and parallel tracheids, interspersed by few medullar rays (purple arrows) ((a), (b)). The resin ducts (black arrows) are associated with late wood (b). In both images, the distinction between early wood (large diameter cells) and late wood (small diameter cells with thick cell walls) is clear.

In general, the anatomical structure of gymnosperm wood is considered simple when compared with angiosperms because it is mainly composed of tracheids, interspersed by radial rays (medullar rays), and some parenchyma cells associated with resin ducts [1] (Figure 2).

The long tapering tracheids constitute the dominant cell type of the conifer axial system and ensure both water conduction and tree support. The rays are responsible for the translocation of water and nutrients between the secondary xylem and the secondary phloem [1].

In temperate regions, the vascular cambium is dormant during winter and reactivates in spring. Reactivation implies that cambial cells take up water and start to expand and divide. Due to expansion, the radial walls of the cambial cells become thinner inducing the peel-off of the bark (all tissues outside the vascular cambium). In Spring, large amounts of secondary xylem and phloem could be harvested [11–13]. Gene expression studies related to wood formation and secondary growth have been based on RNA isolation from these vascular tissues, and a large number of expressed sequence tags (ESTs) have been developed from differentiating xylem of different species.

## 3. Plant Models Used in the Study of the Xylem Transcriptome

Several genetically modified or mutant plant species have been used as model systems for the study of wood formation in trees [14–17]. Among them, *Arabidopsis thaliana* has been considered the best genetic model for the study of xylogenesis [18, 19]. Some mechanisms of cell differentiation are similar and shared among the higher plants, namely, between the primary and secondary vascular tissues. These features allowed judicious extrapolation of some findings achieved in herbaceous plants to the study of secondary growth in trees [18, 20, 21]. Due to moderate conservation of the xylem transcriptome, homolog transcription factors and xylem ortholog genes were found among *Arabidopsis* and diverse *taxa* of vascular plants [6, 22–24].

Although *Arabidopsis* has been considered an excellent genetic model for the study of xylogenesis in trees [18, 19], the following disadvantages were pointed out: (i) reduced plant size; (ii) annual growing habit which disables studies of seasonal variation of xylem differentiation, dormancy, and cambial aging process; and (iii) single cell type [8, 11].

The adaptation of new genetic technologies to forestry species made the use of trees as model plants possible for studies of wood formation and differentiation. *Populus*, *Acacia,* and *Eucalyptus* have been used as models for angiosperms, while pines and spruce constitute excellent models for gymnosperms. Pines have a remarkable variability of wood characteristics, resultant from genetic, environmental, and developmental factors, making them suitable for the identification of candidate genes related to wood quality traits in conifers [11].

## 4. Transcriptome Studies Related to Wood Formation and Quality

The transcriptome is the set of RNA molecules (transcripts) in a given cell or tissue at a particular time-point and condition, representing the transcribed portion of the genome. Transcriptome studies based on different technologies have provided insights into the function and regulation of expressed genes in different conditions (cell type, tissue, stress, etc.). The full characterization of a transcriptome is the key step to understand life diversity, for genome annotation and evaluation of the temporal and spatial patterns of gene expression [25].

Genes encoding for primary and secondary cell wall formation, enzymes of the monolignol biosynthesis, non-cell-wall genes (e.g., transcription factors), and others with unknown function have been related to wood formation and quality.

A large scale of ESTs (expressed products of genes functioning in certain tissues under specific conditions) generated from large numbers of cDNA libraries isolated from specialized tissues and organs have been the main source of gene profiling studies [12, 21]. ESTs have been automatically annotated and processed so quickly that sometimes the inherent information content appears to be underexploited. Some computational, functional, and comparative genomic approaches were reported by [26] in order to improve the uncovering of interesting genes and the annotation of several contigs from pine and other gymnosperm libraries.

The Computational Biology and Functional Genomics Laboratory (at the Dana-Farber Cancer Institute and Harvard School of Public Health) (http://compbio.dfci.harvard.edu/tgi/) developed the Gene Index project which integrates the gene indexes of different organisms based on international research data of EST sequencing and gene research projects. Among the 60 plants listed in this public database, we found out gene indexes for *Pinus*, *Picea*, *Quercus*, and *Populus* (Table 1).

For each EST sequence, the Gene Index project provides information about cellular role, metabolic and signalling pathways functions, and prediction of alternative splice variants, among other features.

### 4.1. Genes Related to Cell Wall Formation and Mechanical Stress

The gene profiling during wood formation revealed expression of several genes associated with the late differentiation stages, including secondary cell wall biosynthesis and cell death (after xylem cell maturation) [27].

Different technologies, including the cDNA sequencing [12, 28, 29], microarrays [4, 30], and serial analysis of gene expression (SAGE) [31], have been helpful for the identification and expression profiling of candidate genes related to the wood formation and quality in conifers. The analysis of differentiating xylem in pines (*Pinus taeda* L. and *Pinus pinaster* Ait.) by cDNA sequencing and microarrays revealed homologues and novel genes related to cell wall proteins, involved in the carbohydrate metabolism, and enzymes involved in the lignin biosynthesis [9, 12, 28, 30]. These proteins play important roles in determining the chemical composition and morphology of the cell wall and consequently wood quality. The differential expression of candidate genes for cell wall formation and lignification arises from differences in protein synthesis and in the rate of cell division in the stem [12], environmental adaptation [30], and seasonal variation [9, 32] verifying that this complex trait is determined by both genetic and environmental factors. The seasonal dynamics of cambial growth in response to climate conditions were also reported for *Pinus halepensis* Miller [32]. Generally, wood could be a biomarker for environmental changes, differences along the growing season, genotype variability, tree age, stages of development, and mechanical stress [6, 32–34].

Wood quality is also largely affected by its mechanical properties, which are determined by the orientation of cellulose microfibrils in secondary cell wall, and mechanical strength [4]. Gene expression profiling by cDNA microarrays developed among *P. radiata* trees revealed that genes involved in cytoskeleton development and secondary cell wall formation (cellulose and lignin biosynthesis) were preferentially transcribed in wood with higher stiffness and low microfibril angle (latewood) [4]. Conversely, genes involved in cell division and primary cell wall synthesis were abundantly transcribed in early wood, which presents low stiffness and high microfibril angle [4]. Juvenile wood has poor quality (low density) due to higher proportion of early wood than that of late wood. The knowledge of genes responsible for the differentiation of late wood (high quality wood) could drive specific breeding strategies based on genetic modification in order to improve the wood quality traits.

Gravitropic response (stem inclination) induces stress and affects wood formation and quality. It has been widely studied in *Eucalyptus*, *Populus trichocarpa,* and pines in order to profile transcripts related to tension and compression wood, and to achieve clues about their regulatory network [27, 35–38]. Sequencing of cDNA samples derived from inclined stems of *P. radiata* and *P. pinaster* and further comparative analysis and validation per quantitative real-time polymerase chain reaction (qRT-PCR) allowed the identification of differentially expressed unigenes involved in hormone regulation, phenylpropanoid pathway, signal transduction, and wood formation [29].

In addition to the identification of genes and their respective functional categories, the gene profiling studies developed so far in gymnosperms and angiosperms revealed differential expression among dissimilar types of wood (early wood *versus* late wood, tension, or compression *versus* normal wood) and enabled insights into their regulatory networks [39]. The understanding of such molecular mechanisms is essential for the improvement of density [5] and other properties related to wood quality.

### 4.2. Genes Involved in the Monolignol Biosynthetic Pathway

**Figure 3:**
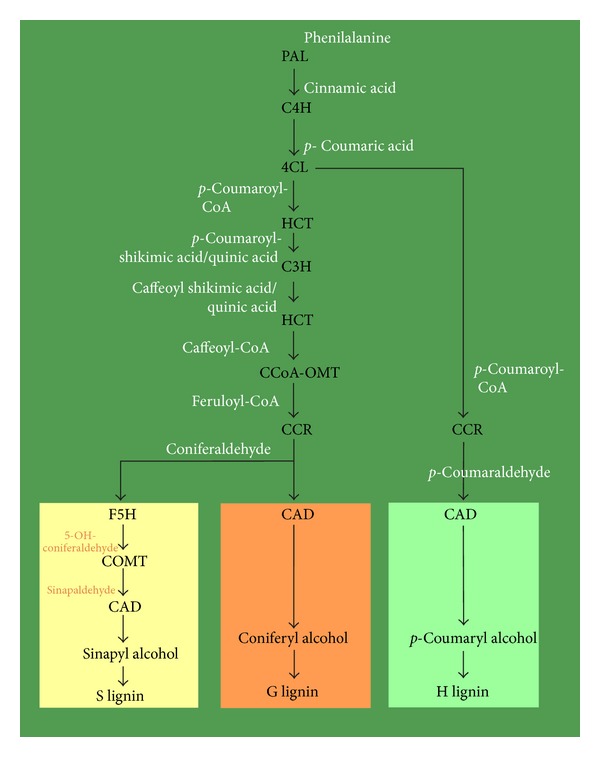
Biosynthetic pathway of the monolignols (monomers of the S-, G- and H-lignin). Adapted from [40, 42]. Substrates of each enzymatic reaction are presented outside the arrows (at white or orange). Enzymes involved in each step are presented as acronyms. PAL phenylalanine ammonia-lyase; C4H; cinnamate 4-hydroxylase; 4CL; 4-coumarate:CoA ligase; C3H; *p*-coumarate 3-hydroxylase; HCT; *p*-hydroxycinnamoyl-CoA:quinate/shikimate *p*-hydroxycinnamoyl-transferase; CCoAOMT; CAFFEOYL-CoA O-methyltransferase; CCR; cinnamoyl-CoA reductase; F5H; ferulate 5-hydroxylase; COMT; caffeic acid O-methyltransferase; CAD; cinnamyl alcohol dehydrogenase.

The major secondary cell wall constituents are cellulose (40–50%), hemicellulose (around 25%), and lignin (25–35%) [8]. Lignin is a heterogeneous phenolic polymer and its monomers (monolignols) are *p*-coumaryl, coniferyl, and sinapyl alcohols (Figure 3). Conifers have the G-lignin type and the coniferyl alcohol is the most abundant monolignol [39, 40]. The monolignols are synthesized in the cytoplasm and translocated to the apoplast to be polymerized into lignin [41].

The monolignol biosynthetic pathway is complex (Figure 3) and has been revised and updated during the last decade [17, 27, 40, 42].

Most of the enzymes and corresponding genes involved in the monolignols biosynthetic pathway have been identified [17, 27]. Hence, it is possible to ascribe a modified wood quality trait to a particular mutation, genetic modification, or differential expression of candidate genes involved in the lignin biosynthesis. In *P. taeda*, the occurrence of a sequence mutation in the CAD gene (*cad-n1*) causes a deficiency in the production of cinnamyl alcohol dehydrogenase (CAD), inducing altered lignification, differences at wood density, and growth [43, 44], affecting its quality. Candidate genes such as those encoding for the enzymes (4CL, C4H, C3H, and CcOAOMT) also matched with Quantitative Trait* Loci *(QTL) for wood density in *P. taeda *[45]. The same technique revealed a positional candidate gene—*KORRIGAN* (involved in hemicellulose and cellulose biosyntheses)—which was mapped with wood-quality-related QTLs in *P. pinaster* [46, 47]. The candidate genes approach based on QTL mapping [48] and gene modification have been useful for wood formation and quality studies. The silencing of the *HCT* enzyme and the suppression of 4-coumarate-CoA ligase (4CL) in *P. radiata* reduced the lignin content, affected its structure, and induced changes in the wood-bark ratio [49, 50]. Pine xylogenesis seems to depend greatly on an adequate supply of lignin “building blocks” (lignin precursors) [50]. The phenotypic and structural changes induced by gene silencing could explain the metabolic plasticity of the lignification process which induces high variation in the lignin content and composition among species, within species, cell types, tissues, developmental phases, seasons, and/or environmental conditions [8, 12, 17, 33, 49, 51].

## 5. Regulatory Mechanisms of Secondary Growth and Wood Formation

The extensive production and analysis of ESTs from wood-forming tissues have revealed a differential and coordinated expression of genes coding for cell wall structural proteins and enzymes associated with the biosynthesis of secondary cell wall polysaccharides (e.g., cellulose), degradation and modification of primary cell walls, biosynthesis of lignin precursors, polymerization of lignin in secondary walls, and programmed cell death [21, 52–54]. The coordinated expression of these genes has reflected an interaction of regulatory mechanisms involving specific transcription factors information molecules of the cell wall microRNAs and phytohormones [13, 21, 53, 55].

### 5.1. Regulation by Transcriptional Factors

Despite the striking correlation among different types of regulation processes, major importance has been given to the regulation by specific transcription factors that are preferentially expressed during wood formation [13, 21]. Transcription is more tractable for study given technologies such as qRT-PCR, cDNA microarrays, high-throughput sequencing, visualization of expression patterns by *in situ *hybridization, candidate genes approach, and new computational methods to unravel complex transcriptional networks [13]. These technologies allowed the functional characterization of several classes of transcription factors, including those which regulate meristem maintenance, tissue differentiation, cell differentiation, and secondary cell wall synthesis, contributing to the understanding of their regulation roles in secondary growth [13, 21].

The R2R3-MYBs constitute one of the largest families of transcription factors which regulate the lignification cell differentiation organ identity and cell fate in angiosperms and conifers such as *P. taeda* and spruce [36, 51, 53, 56–58]. Overexpression of MYB factors in transgenic plants induced reduced expression of lignin biosynthetic genes and decrease in lignin content [51]. The MYB1 and MYB8 factors seem to be part of a conserved transcriptional network involved in secondary cell wall deposition in conifers [58].

The NAM/ATAF/CUC (NAC) family genes have been widely studied in angiosperms and are preferentially expressed in developing vascular tissues, being responsible for the secondary cell wall thickening and fibre differentiation [21]. The transcriptional regulation by NAC factors is conserved among *Arabidopsis* and trees, but it evolved to a more complex regulatory network in the forestry species [22–24]. The poplar wood-associated NAC domain transcription factors (*PtrWNDs*) also activate a set of downstream transcription factors, and together, they coordinate the regulation of secondary wall biosynthesis [23, 59].

The class III homeodomain-leucine zipper (HD-ZIP III) transcription factors and the KANADI (KAN) genes transcription factor family have overlapping and antagonistic roles in the regulation of wood formation [21, 52].

### 5.2. Transcriptional Regulation by Phytohormones

The activation of cambial growth, cell division, cell extension in the stems, and the formation of lateral roots by low concentrations of pure hormones were demonstrated early in herbaceous plants [60]. More recent studies have illustrated the transcriptional regulatory role of phytohormones in the cell wall dynamics, wood formation, and quality [53, 55, 61–63]. This regulatory mechanism is mediated by the expression of specific genes and numerous transcription factors which control the cambium activity and secondary growth [21, 53]. Streams of hormonal signals such as auxins, gibberellins, cytokinins, and ethylene are synthesized in different locations and move through the vascular tissues [62, 63]. The phytohormonal mechanisms that control wood quality and formation are very well documented in [63]. The indole-3-acetic acid (IAA) is the most naturally occurring auxin. It is produced in the young leaves, and it moves downward through the cambium to the root tips and constitutes the major hormonal signal which regulates wood formation, by controlling the cambial activity and inducing the xylem and phloem differentiation. Along the tree axis, there are variable concentrations of auxin that influence cell width, wall composition, and wood density [63, 64]. Asymmetric hormonal distributions can promote the formation of compression wood in conifers and tension wood in angiosperms. The cytokinins (Cks) are adenine derivatives, produced in the root caps, and move upward, stimulating cell divisions in the vascular cambium and increasing its sensitivity to the auxin signal. The gibberellins (GAs) are a large family of tetracyclic diterpenes which promote cell and stem elongation, inducing long tracheids (in gymnosperms) and fibres (in angiosperms), and they regulate the lignin biosynthesis and the transition from juvenile to mature wood. Exogenous applications of GAs in young conifers could accelerate their reproductive phase and cone production. Ethylene (C_2_H_4_) is synthesized in response to stress (wounding, flooding wind bending, high auxin and Cks levels). When highly concentrated, it inhibits stem elongation and promotes leaf and fruit abscission. In conifers, the ethylene mediates the methyl jasmonate-defence-response by inducing traumatic-resin ducts, which, in high number, negatively affect the wood quality. The abscisic acid (ABA) is the universal stress hormone, present in all higher plants, and it plays a central role in the plasticity of plant development, once it could slow down and stop the wood formation by retarding or ending the cambial activity during winter [63].

The secondary xylem development is also controlled by a crosstalk among different plant hormones [55, 65]. The involvement of phytohormones in the posttranscriptional regulation of the secondary xylem development was also reported by [13, 54, 55].

### 5.3. Posttranscriptional Regulation by miRNAs

Ample interest has been driven in the last years to the study of miRNAs. These small (~22 nt) double stranded noncoding RNAs derive from intergenic regions of the genome and are posttranscriptional regulators of endogenous genes. The miRNAs induce gene silencing by cleavage or repression of the messenger RNA (mRNA) [66–69]. Their regulatory activities have been demonstrated by their targets identification, by using physiological and phenotypic assays, bioinformatics, genomics, and biochemical tools. The effective levels of plant miRNAs are determined by transcription, processing, miRNA-induced silencing complex loading, turnover, and decay. Each process is affected by factors such as genomic modifications, RNA editing, miRNA-induced silencing complex loading competition, target abundance and complementarities, and spatial-temporal effects, conferring a highly dynamic feature to miRNA activities (see revision of [70]). Generally, miRNAs play important regulatory roles in plant development, growth, defence, response to stress (biotic, abiotic, and mechanical), and adaptation to environmental changes [27, 71–74]. They are also involved in the coordinated regulatory mechanisms of secondary growth and wood formation by targeting phytohormones and transcription factors [27, 71, 75–77] (Table 2).

Considering the predicted functions of some miRNA families presented in Table 2, their targeting of transcription factors (e.g., MYB, NAC-domain protein, Class III HD-Zip protein) and phytohormones (e.g., auxin-responsive factor; auxin/IAA) involved in the regulation of wood formation [27] is clear. 

Some of the miRNA families identified in poplar were conserved in *Arabidopsis*, *P. taeda, *or *Pinus densata* (Table 2). Recently, a mRNA library derived from *P. densata *needles was sequenced by the Illumina high-throughput sequencing technology, and it allowed the identification of 34 miRNA families that were conserved in *A. thaliana*, *Oryza sativa*, poplar, *Vitis vinifera*, *P. taeda,* and *Picea abies* [78]. The *miR156*, *miR159*, *miR160,* and/or *miR171* families are conserved in poplar, *Arabidopsis*, *P. taeda,* and/or *P. densata* and present similar functions among them (Table 2). These four miRNA families belong to a group of 21 elements, considered to be the most ancient miRNAs, due to their conserved expression among several angiosperms, gymnosperms, lycopods, and bryophytes [79].

The ongoing actualization of miRNA databases such as miRBase (http://www.mirbase.org/), PMRD—plant microRNA database (http://bioinformatics.cau.edu.cn/PMRD), and MIRNEST version 1.0 (http://lemur.amu.edu.pl/share/php/mirnest/home.php) includes stem-loop, mature miRNA sequences, and target predictions. The last releases of these databases presented several miRNA sequences for the following gymnosperm species: *Cycas rumphii* (1 miRNA sequence, *cru*-MIR156), *P. taeda* (139 miRNA sequences), *P. pinaster *(8 miRNA sequences),* P. densata *(31 miRNA sequences), *Pinus contorta* (6 miRNA sequences), *Pinus banksiana* (13 miRNA sequences), and/or *Picea *sp. (49 miRNA sequences). Inside the MIRNEST database, we also find the MicroPC resource tool which enables the prediction and the comparison among plant miRNAs (http://www3a.biotec.or.th/micropc/), despite the existence of other online tools for the same purpose.

The identification of key transcription factors and phytohormones targeted by miRNAs will provide the knowledge of binding sites for specific and crucial genes expressed during wood formation enabling the establishment of an initial transcriptional network.

### 5.4. Cell Wall Regulation of Secondary Growth

During the differentiation within secondary xylem and phloem, the stem cell walls are extensively modified. In angiosperms, the differentiation of tracheary elements and fibers involves the synthesis of a lignified secondary cell wall between the primary cell wall and the plasma membrane. Studies realized in poplar, *Arabidopsis* and *Zinnia*, have demonstrated that proteins involved in the generation of wall-derived signals or cell wall modifications, such as chitinase-like enzymes, contribute to developmental mechanisms [53]. The arabinogalactan proteins (AGPs)—highly glycosylated proteins of the cell walls—have been considered as putative substrates of chitinases. These proteins are expressed in vascular differentiating tissues and they are upregulated during tension wood formation, and they regulate the differentiation of tracheary elements by inductive cell-cell interaction [80, 81].

### 5.5. Interacting Regulatory Mechanisms

The complex developmental processes of secondary growth and xylogenesis require the interplay of the individual regulatory mechanisms presented here. For instance, different classes of transcriptional factors directly regulate genes that encode biosynthetic enzymes for cell wall or phytohormones. The cell wall dynamics are regulated by phytohormones such as auxin, and the lignification of the stem cell walls is controlled by gibberellins (GAs). In addition, auxin-related genes, KAN, and HD-ZIP III transcription factors are posttranscriptionally regulated by miRNAs [21, 53, 54, 82]. The intricate correlation among these regulatory mechanisms is partially understood in trees, but we are confident that the availability of new genomic technologies and the whole genome sequencing of conifer species will provide, in the near future, the establishment of transcriptional network for secondary growth and wood formation.

## 6. The “Next-Generation Genomics” for Conifers

A number of next generation sequencing (NGS) technologies have emerged in the past few years. The Roche 454 FLX system, Illumina, and Life Tech SOLiD are now referred to as second generation sequencing (SGS) platforms. The most recently developed technologies, such as single-molecular real-time (SMRT) sequencer, Heliscope Single Molecule Sequencer, and the Ion Personal Genome Machine, are considered the third-generation sequencing (TGS) [83, 84]. The TGS generate-longer sequence reads in a shorter time and with lower costs per instrument run, compared to the SGS. The accessibility to both SGS and TGS technologies, with ever declining costs and increased data output, will enable the plant genomics and breeding community to undertake genotyping by sequencing (GBS) [84]. The SGS has been used for *de novo* sequencing, genome resequencing, and whole genome and transcriptome analysis [25, 83–87].

The combination of gene data provided by sequencing, expression profiling, and protein analyses could establish the linkage between genotype, gene function, and phenotype [13, 25, 87]. The knowledge of this complex relationship and the highly conserved xylem transcriptome among conifers [6] will enable the construction of regulation models for secondary growth and wood formation and the designing of strategies for the improvement of wood production and quality traits, involving reduced breeding cycles and increased selection accuracy [13, 87].

In the past few years, several initiatives have been launched for the sequencing of whole conifer genomes, including pines, spruces, and Douglas-fir [87]. Genome-wide analysis of conifers is particularly challenging due to their largest genome size (ranging from 20 to 30 gigabases), outcrossing mating system, wind-pollinated, very large effective population size, and high heterozygosis [88]. However, massively parallel DNA sequencing data was used to assemble a draft of the 20-Gb nuclear genome of Norway spruce (*Picea abies* (L.) Karst) [88]. Later, the assembly of the 20.8-Gb *Picea glauca* (white spruce) genome from whole-genome shotgun sequencing data was reported [89]. The draft assembly of the *P. abies* genome provided a deep characterization at the structure and content levels, as well as new insights into the gymnosperms evolution [88]. The coding and noncoding fractions of this conifer genome were compared with the low-coverage draft genome assemblies of five other gymnosperms. Once there was no evidence of recent whole genome duplications (WGDs), the large genome size was explained as resulting from a slow and steady accumulation of long terminal repeat (LTR)–retrotransposons (RTNs), due to the lack of an efficient mechanism of elimination. The diversity of transposable elements (TEs) was shared among the six gymnosperms, being the LTR-RTNs the most abundant class. Considering their two major super-families, in the six gymnosperms, the *Ty3/Gypsy* was more abundant than the *Ty1/copia*. The 24-nucleotide small RNAs, known to be implicated in the silencing of TEs by the establishment of DNA methylation, showed a tissue-specific expression and reduced levels. Numerous long introns (>10,000 bp), gene-like fragments, uncharacterized long noncoding RNAs, and short RNAs were also found [88]. The large size of the introns suggested a very early expansion on the evolutionary story of conifers probably due to TEs insertion [88].

The sequencing of the whole *Picea *sp. genomes, which are two ecologically and commercially important species, opened up new windows for research, forestry, and breeding. These assemblies will enable future identification and study of gymnosperm genes, the assistance of forest management strategies, and the understanding of the environmental and biological interactions of spruce trees [89].

## 7. Concluding Remarks and Perspectives

Conifers are dominant in the northern hemisphere forests. They present ecological and commercial important roles, particularly due to wood production. Nowadays, the global climatic changes have been threatening the surviving and production rates of several plant species, including the conifers, which face deforestation problems. In order to partially overcome these problems, forestry researchers have tried to deeply understand the molecular mechanisms involved in stress, wood formation, and quality. As reported by [13], cambium is one of the most important, but the least understood, plant meristem, and it is an exciting time to study secondary growth due to the availability of well-suited new genomic technologies. Comparative genomics among vascular nontree species, angiosperm trees, and conifers has contributed to the partial understanding of secondary growth and wood formation, given the moderate conservation of candidate genes, transcription factors, and regulatory mechanisms among them. However, the interacting regulatory mechanisms are partially unknown [57]. In the last decades, the genomics and transcriptomics knowledge has evolved faster for angiosperms than for gymnosperms, even more, after the whole genome sequencing of poplar. However, we are confident that the recent draft assemblies of the whole genomes of *P. abies* and *P. glauca* will change that feature, by contributing to the establishment and understanding of the transcriptional and regulatory networks underlying the secondary growth and wood formation in conifers. Along with the fundamental research and knowledge about the evolution and developmental biology of the secondary growth, these discoveries will have practical applications under the scope of genetic improvement of wood production and quality traits.

## Figures and Tables

**Table 1:** Last releases relative to the number of inputs (ESTs) and output sequences (EST, ET, and TC) per forestry genus available on the Gene Index project database.

Genus	Input sequences	Unique output sequences		
	ESTs	ESTs	ETs^1^	TCs^2^
*Quercus* (OGI project)	148,876	22,470	0	19,674
*Pinus* (PGI project)	452,256	32,337	131	44,858
*Populus* (PplGI)	423,556	50,065	248	50,563
*Picea* (Sgi project)	541,490	34,866	26	44,517

^1^ETs: nonredundant transcripts (contain a set of nucleotide sequences that represent mature transcripts); ^2^TCs: tentative consensus sequences that are created by assembling ESTs into virtual transcripts, and they could be based on two or more ESTs (and possibly an ET) that overlap for at least 40 bases with at least 94% of sequence identity. They could comprise ESTs derived from different tissues.

**Table 2:** Specific and conserved miRNA sequences identified in angiosperms and/or conifers and respective predicted functions or targets. Some of the targets correspond to hormones and transcription factors involved in the secondary growth and wood formation.

	miRNA	Predicted functions or targets
Conserved miRNAs between poplar and *Arabidopsis* [25, 66]	*ptr-miR156 *	(i) SPB-like (ii) Nitrate transporter
	*ptr-miR159 *	(i) MYB (ii) Asparagine synthase (iii) (1-4)-b-mannan endohydrolase
	*ptr-miR160 *	Auxin-responsive factor
	*ptr-miR162 *	DCL1
	*ptr-miR164 *	(i) NAC-domain protein (ii) Protein kinase
	*ptr-miR168 *	(i) Vesicle coat protein complex COPI (ii) AGO1
	*ptr-miR171 *	Scarecrow-like transcription factor
	*ptr-miR172 *	Homeotic protein APETALA2
	*ptr-miR319 *	MYB
	*ptr-miR408 *	(i) Plastocyanin-like (ii) Early-responsive to dehydration-related protein
	*ptr-miR472 *	Putative disease resistance protein

Poplar miRNAs [66]	*ptr-miR473 *	(i) UV-B-resistant protein (UVR8) (ii) GRAS domain-containing protein
	*ptr-miR474 *	(i) PPR (ii) Protein kinase (iii) Kinesin (iv) Leucine-rich repeat
	*ptr-miR475 *	PPR
	*ptr-miR476 *	PPR
	*ptr-miR477 *	(i) GRAS domain-containing protein (ii) NAC-domain protein (iii) Zinc finger protein
	*ptr-miR478 *	Organic anion transporter
	*ptr-miR480 *	Proton-dependent oligopeptide transport family protein
	*ptr-miR482 *	Putative disease resistance protein

* P. taeda* miRNAs [67]	*pta-miR156 *	(i) Peptidyl-tRNA hydrolase-like (ii) SPB-domain protein
	*pta-miR159 *	(i) MYB (ii) Programmed cell death 6 protein-like
	*pta-miR160 *	(i) Auxin-responsive factor 10 (ARF10) (ii) Aux/IAA protein
	*pta-miR319 *	Acyl-ACP thioesterase
	*pta-miR946 *	Disease resistance protein
	*pta-miR947 *	(i) Pepsin A (ii) Microtubule-bundling polypeptide (iii) Non-protein-coding genes
	*pta-miR948 *	(i) Serine/threonine kinase (ii) Pepsin A
	*pta-miR950 *	(i) Non-protein-coding genes (ii) AMP-binding protein
	*pta-miR951 *	Non-protein-coding genes
	*pta-miR952 *	(i) Multidrug resistance-associated protein (ii) Thaumatin-like

Conserved miRNAs found in *Pinus densata* [70]	*pde-miR162 *	(i) DCL1 (RNA processing) (ii) Nodal modulator 1-like (carboxypeptidase activity)
	*pde-miR166 *	Class III HD-Zip protein HDZ33 (DNA binding)
	*pde-miR171 *	(i) GRAS family transcription factor (DNA binding) (ii) Actin binding protein (actin binding)
	*pde-miR482 *	Histone deacetylase (histone deacetylation)
	*pde-miR2118 *	CC-NBS-LRR resistance-like protein (defence response)
